# Maternal hookworm modifies risk factors for childhood eczema: results from a birth cohort in Uganda

**DOI:** 10.1111/pai.12251

**Published:** 2014-08-29

**Authors:** Harriet Mpairwe, Juliet Ndibazza, Emily L Webb, Margaret Nampijja, Lawrence Muhangi, Barbara Apule, Swaib Lule, Hellen Akurut, Dennison Kizito, Mohammed Kakande, Frances M Jones, Colin M Fitzsimmons, Moses Muwanga, Laura C Rodrigues, David W Dunne, Alison M Elliott

**Affiliations:** 1 MRC/UVRI Uganda Research Unit on AIDSEntebbe, Uganda; 2 London School of Hygiene and Tropical MedicineLondon, UK; 3 Entebbe HospitalEntebbe, Uganda; 4 Department of Pathology, University of CambridgeCambridge, UK

**Keywords:** birth cohort, children, eczema, effect modification, hookworm, IgE, incidence, pregnancy, skin prick test, Uganda

## Abstract

**Background:**

Worms may protect against allergy. Early-life worm exposure may be critical, but this has not been fully investigated.

**Objectives:**

To investigate whether worms in pregnancy and in early childhood are associated with childhood eczema incidence.

**Methods:**

The Entebbe Mother and Baby Study, an anthelminthic treatment trial, enrolled pregnant women between 2003 and 2005 in Uganda. Mothers were investigated for worms during pregnancy and children annually. Eczema was doctor-diagnosed from birth to age five years. A planned observational analysis was conducted within the trial cohort to investigate associations between worms and eczema.

**Results:**

Data for 2345 live-born children were analysed. Hookworm was the most prevalent maternal worm (45%). Childhood worms were less prevalent. Eczema incidence was 4.68/100 person-years. Maternal hookworm was associated with reduced eczema incidence [adjusted hazard ratio (95% confidence interval), p-value: 0.71(0.51–0.99), 0.04] and modified effects of known risk factors for eczema: Dermatophagoides-specific IgE in children was positively associated with eczema incidence if the mother had no hookworm [2.72(1.11–6.63), 0.03], but not if the mother had hookworm [0.41(0.10–1.69), 0.22], interaction p-value = 0.03. Similar interactions were seen for maternal history of eczema {[2.87(1.31–6.27, 0.008) vs. [0.73(0.23–2.30), 0.60], interaction p-value = 0.05}, female gender {[1.82(1.22–2.73), 0.004 vs. [0.96(0.60–1.53), 0.87], interaction p-value = 0.04} and allergen-specific IgE. Childhood*Trichuris trichiura* and hookworm were inversely associated with eczema.

**Conclusions:**

Maternal hookworm modifies effects of known risk factors for eczema. Mechanisms by which early-life worm exposures influence allergy need investigation. Worms or worm products, and intervention during pregnancy have potential for primary prevention of allergy.

Allergy is a global problem affecting approximately 20% of the world's population (1) and is on the increase in developing countries, but the causes of this increase are unknown (2). Environmental factors and gene–environment interactions have an important role in the aetiology of allergy (3).

Atopy, measured by increased allergen-specific IgE or positive skin prick responses to allergens, is a strong risk factor for allergy in developed countries but reportedly less so in developing countries (4–6). The reasons for this discrepancy are not known, but there is a suggestion that chronic immune-modulating infections such as worms, which are prevalent in developing countries, could play a role (7). Several observational studies have investigated associations between worms and allergy [reviewed by Leonardi-Bee et al. (8) and Flohr et al. (9)]. Results from these studies are inconsistent: some show an inverse association, others a positive association and some no association. There is evidence that prenatal exposure to worms influences later responses to childhood worm infections (10) and possibly to unrelated antigens such as BCG vaccine (11). In this analysis, we investigated whether exposure to worms during pregnancy and in early childhood was associated with eczema incidence in early childhood.

## Methods

### Study setting

Entebbe Mother and Baby Study (EMaBS) was a trial among pregnant women and their offspring residing in peri-urban, fishing and rural communities beside Lake victoria, Uganda. Details of the trial design are reported elsewhere (12). In summary, EMaBS was a randomized, double-blinded, placebo-controlled trial of anthelminthic treatment in pregnancy using single-dose albendazole (400 mg) or placebo and praziquantel (40 mg/kg) or placebo in a 2 × 2 factorial design. At age fifteen months, the children resulting from these pregnancies were randomized to receive either albendazole or placebo quarterly until age five years (12).

The study was approved by the Uganda Virus Research Institute Science and Ethics Committee, the Uganda National Council for Science and Technology, and the London School of Hygiene and Tropical Medicine ethics committee.

### Specific objective

The objective of this analysis was to investigate whether worm infections in pregnancy and in early childhood are associated with reduced eczema incidence in the first five years of life.

### Study enrolment and follow-up

EMaBS eligibility criteria have been reported elsewhere (12). Eligible pregnant women provided written informed consent prior to enrolment and provided stool and blood samples at enrolment and delivery. At enrolment, a questionnaire was completed, and women underwent testing for asymptomatic malaria and for HIV. Six weeks after delivery, all women received anthelminthic treatment with both albendazole and praziquantel. They were encouraged to bring the children to the study clinic for routine immunisations and for the treatment of illnesses, all free of charge.

Children were routinely seen annually, as close to their birthday as possible, to provide stool and blood samples. Worm infections detected at annual visits were treated. Children seen at age three between November 2007 and March 2009 were selected for skin prick tests and allergen-specific IgE testing.

### Skin prick tests and allergen-specific IgE

We conducted skin prick testing (SPT) according to standard procedure (13) using*Blomia tropicalis*, Dermatophagoides mix, cow's milk and egg white, with histamine and saline controls (ALK Abello, Hoersholm, Denmark). A diameter of ≥3 mm was considered positive.

Allergen-specific IgE testing was performed for children who underwent SPT at three years by ELISA, as previously described (14), using Dermatophagoides mix (*Dermatophagoides farina* and*Dermatophagoides pteronyssinus*) and German cockroach (*Blattella germanica*) (Greer Labs, Lenoir, NC, USA). Positive sera standardized using the Immunocap Assay System (Phadia, Uppsala, Sweden) were used to standardize the ELISA assay.

### Other laboratory procedures

Stool samples were analysed by the Kato-Katz (15) method. Two slides were examined for each sample, read within 30 min for hookworm and the following day for the other worms including*Schistosoma mansoni*,*Trichuris trichiura* and*Ascaris lumbricoides*. We used the charcoal culture method (16) for*Strongyloides stercoralis,* thick blood smears for malaria infection and the modified Knott's method for*Mansonella perstans* and other microfilariae (17).

### Statistical methods

Data were collected on pre-coded case report forms and analysed using stata version 10 (Stata Corp., College Station, TX, USA). The sample size of 2500 pregnant women for the EMaBS trial was planned for infectious disease end-points for the trial interventions (12). Assuming eczema incidence of 5 per 100 person-years, this observational analysis would have over 80% power to detect exposures with a rate ratio of 1.30 or higher, at 5% significance level. This observational analysis was a planned secondary analysis for the trial cohort.

#### Outcomes and exposures

The primary outcome for this analysis was doctor-diagnosed eczema, defined as a recurrent itchy rash, with either wet weeping skin or dry scaly skin and with a typical distribution (18). Eczema was either a presenting complaint or an incidental finding during routine or other illness visits.

Worm infections were the primary exposures of interest. Maternal socio-economic status, education, area of residence and history of allergy were considered as potential confounders for the association between worms and eczema and as exposures of interest in their own right. In addition, children's birth order, birth weight and childhood illnesses were considered as potential confounders for the association between childhood worms and eczema, as well as exposures. Children's atopic status (SPT and allergen-specific IgE) was considered as exposures.

For atopy and for other factors that were positively associated with eczema, maternal hookworm was investigated as a potential effect modifier. Effect modification by the trial interventions was also investigated.

#### Recoding of variables

Principal component analysis was used to identify dominant variables (household items owned, building materials and number of rooms) to create a score that was regrouped as lower vs. higher socio-economic status (19). Area of residence was divided into zones based on topography and the grouping of settlements, which followed geo-referencing of participant's addresses using hand-held global-positioning system receivers (Garmin eTrex) (20). A World Health Organisation (WHO) infection intensity classification was used for hookworm (21). For maternal asymptomatic malaria, results at enrolment and delivery were combined to generate a binary variable for malaria during pregnancy.

For childhood illnesses, numbers of episodes of malaria, diarrhoea, lower and upper respiratory tract infection were analysed as continuous variables. Due to the low prevalence of childhood worm infections, data from one to five years were combined into a single binary variable (ever infected or never infected) for each type of worm infection. We used the standard cut-off point (0.35 kUA/l) for allergen-specific IgE to categorize children as atopic or non-atopic.

#### Regression analysis

We used Cox regression to assess crude and adjusted associations between exposures and eczema incidence, with robust standard errors to allow for clustering of events within each child. The Wald test was used to test for effect modification. For each child, time at risk began at birth and ended either at five years of age, loss to follow-up or death. Factors listed above as potential confounders were included in multivariable models, with the exception of those with substantial missing data and those whose inclusion had no impact on the hazard ratios for other exposures. Analyses for child's gender and atopy were not adjusted as no potential confounders were identified.

## Results

### Participants' profile

Between April 2003 and November 2005, EMaBS enrolled 2507 pregnant women resulting in 2345 recorded live births. Follow-up of children started from birth of the first child in April 2003 and finished in April 2011, when the last child reached five years. Follow-up time for 116 (5.0%) and 594 (25.3%) of the 2345 live-born children was censored at death or loss to follow-up, respectively, leaving 1635 (69.7%) still in follow-up at age five years (22). The mean number of routine or illness clinic visits per child was 14 (range 0–63).

Mothers were mostly of low education status, and worm prevalence was high (68% infected with at least one worm species).

Eczema incidence was 10.4/100 person-years of follow-up (pyrs) in infancy (14) and 4.68/100 pyrs in the first five years of life.

#### Maternal factors associated with childhood eczema

Associations between maternal factors and eczema incidence are shown in Table1. Children from rural areas had significantly less eczema compared with children from urban areas. Maternal history of eczema was strongly associated with childhood eczema. There was a strong association between maternal albendazole and childhood eczema as previously reported (14).

**Table 1:** Maternal factors and their association with childhood eczema in the first five years of life

Maternal factor	Number of children in each category (%)	No of eczema events	Person-years at risk (×100)	Rate (per 100 pyrs)	Crude HR (95% CI)	aHR*(95% CI)	p-value
Education†							
None/primary	1278 (55)	232	51.65	4.49	1	1	0.85
Post-primary	1063 (45)	220	44.63	4.93	1.11 (0.84–1.54)	0.97 (0.70–1.35)	
Household socio-economic status‡							
Lower status	1053 (46)	164	42.78	3.83	1	1	0.08
Higher status	1248 (54)	274	51.89	5.28	1.39 (0.99–1.96)	1.36 (0.96–1.92)	
Area of residence§							
Entebbe, town	950 (41)	251	38.74	6.48	1	1	0.002
Kiggungu, fishing village	261 (11)	18	11.25	1.60	0.25 (0.14–0.43)	0.27 (0.15–0.46)	
Manyago and Kabale, peri-urban	651 (28)	131	26.89	4.87	0.75 (0.53–1.07)	0.76 (0.52–1.10)	
Katabi, rural	451 (20)	48	18.48	2.60	0.40 (0.25–0.66)	0.42 (0.25–0.68)	
Regular cigarette smoking by adult in house¶							
No	1429 (81)	320	66.18	4.83	1	1	0.56
Yes	330 (19)	81	14.98	5.41	1.11 (0.64–1.92)	1.17 (0.69–1.99)	
Maternal history of asthma∥							
None	1706 (97)	382	78.66	4.86	1	1	0.26
Yes	60 (3)	19	2.84	6.70	1.40 (0.73–2.70)	1.44 (0.76–2.74)	
Maternal history of eczema**							
None	1710 (97)	374	78.94	4.74	1	1	0.02
Yes	56 (3)	27	2.55	10.59	2.24 (1.12–4.47)	2.28 (1.14–4.53)	
Maternal albendazole trial arm during pregnancy							
Placebo arm	1175 (50)	175	48.21	3.63	1	–	0.005
Albendazole arm	1170 (50)	277	48.26	5.74	1.58 (1.15–2.17)		
Maternal worm infections during pregnancy							
1. Hookworm††: None	1311 (56)	298	54.34	5.48	1	1	0.04
Yes	1025 (44)	151	41.78	3.61	0.65 (0.48–0.90)	0.71 (0.51–0.99)	
2*. M. perstans*‡‡: None	1841 (79)	389	75.85	5.13	1	1	0.10
Yes	496 (21)	63	20.32	3.10	0.60 (0.39–0.92)	0.70 (0.45–1.07)	
3*. S. mansoni*††: None	1915 (82)	381	78.56	4.85	1	1	0.51
Yes	421 (18)	68	17.55	3.87	0.80 (0.55–1.15)	0.88 (0.61–1.28)	
4.*S. stercoralis*§§: None	2041 (88)	380	84.03	4.52	1	1	0.33
Yes	283 (12)	65	11.60	5.60	1.23 (0.77–1.95)	1.27 (0.78–2.06)	
5.*T. trichiura*††: None	2130 (91)	422	87.81	4.81	1	1	0.59
Yes	206 (9)	27	8.30	3.25	0.67 (0.24–1.86)	0.73 (0.23–2.33)	
6.*A. lumbricoides*††: No	2282 (98)	466	93.87	4.75	1	1	0.08
Yes	54 (2)	3	2.25	1.33	0.28 (0.09–0.87)	0.37 (0.12–1.14)	
Hookworm category††							
None	1311 (56)	298	54.34	5.48	1	1	0.03
Mild	872 (37)	134	35.70	3.75	0.68 (0.49–0.95)	0.74 (0.53–1.04)	
Mod-severe	153 (7)	17	6.08	2.79	0.51 (0.27–0.96)	0.56 (0.30–1.07)	
Maternal asymptomatic malaria during pregnancy¶¶							
None	2037 (88)	414	84.23	4.91	1	1	0.03
Yes	266 (12)	34	10.64	3.20	0.65 (0.41–1.02)	0.59 (0.37–0.95)	

Pyrs, person years; CI, confidence interval; HR, hazard ratio; aHR, adjusted hazard ratio.

* Adjusted for maternal education, area of residence and household socio-economic status.

No of missing variables:

† = 4,

‡ ‡ = 44,

§ = 32,

¶ = 586,

∥ = 579,

** = 579,

†† = 9,

‡‡ = 8,

§§ = 21,

¶¶ = 42.

Maternal hookworm was inversely associated with eczema incidence, and this association was strongest among those with heavier infections (Table1). In adjusted analyses, maternal infections with other worm species were not associated with childhood eczema. Maternal asymptomatic malaria infection during pregnancy had a strong inverse association with childhood eczema.

#### Childhood factors associated with childhood eczema

As presented in Table2, neither child's birth order nor birth weight was associated with childhood eczema. Female children had a higher eczema incidence. There was an inverse association between the number of malaria episodes and eczema, but not for diarrhoea, lower or upper respiratory infections. The prevalence of worm infections in the first five years was relatively low. Childhood*T. trichiura* and hookworm were inversely associated with eczema. The remaining worm infections showed either a weak inverse association or no association with eczema.

**Table 2:** Childhood factors associated with eczema in the first five years of life

Childhood factor	Number of children in each category (%)	No of eczema events	Person-years at risk (×100)	Rate (per 100 pyrs)	Crude HR (95% CI)	aHR† (95% CI)	p-value*
Child's birth order							
First born	622 (27)	108	24.25	4.45	1	1	0.06
2nd–4th born	1342 (57)	257	55.91	4.60	1.05 (0.72–1.53)	1.07 (0.73–1.58)	
5th born or more	381 (16)	87	16.31	5.33	1.22 (0.73–2.05)	1.37 (0.82–2.27)	
Child's birth weight¶							
Normal	1719 (90)	368	71.70	5.13	1	1	0.48
Low (≤2.5 kg)	187 (10)	31	7.50	4.13	0.81 (0.43–1.51)	0.79 (0.41–1.51)	
Child's sex∥							
Male	1208 (52)	193	49.75	3.88	1	–	0.03
Female	1135 (48)	259	46.72	5.54	1.43 (1.04–1.97)	–	
Common childhood infections diagnosed prospectively (0–5 yr)							
1. Malaria							
Mean (range) of episodes: 1.4 (0–16)	–	–	–	0.94 (0.88–1.00)	0.88 (0.83–0.94)		<0.0001
2. Diarrhoea							
Mean (range) of episodes: 2.6 (0–22)	–	–	–	1.10 (1.05–1.16)	1.02 (0.93–1.10)		0.71
3. Lower respiratory infections							
Mean (range) of episodes: 0.4 (0–9)	–	–	–	1.25 (1.11–1.41)	1.10 (0.96–1.26)		0.18
4. Upper respiratory infections							
Mean (range) of episodes: 11 (0–58)	–	–	–	1.04 (1.03–1.06)	1.00 (0.95–1.04)		0.92
Childhood worm infections‡ (assessed annually from 1 to 5 yr)							
1.*T. trichiura* (810):None	643 (79)	179	32.08	5.58	1	1	0.002
Yes	167 (21)	16	8.13	1.97	0.35 (0.18–0.66)	0.35 (0.18–0.67)	
2.*A. lumbricoides* (765): None	680 (89)	180	33.93	5.30	1	1	0.22
Yes	85 (11)	12	4.11	2.92	0.54 (0.28–1.06)	0.64 (0.32–1.30)	
3*. S. mansoni* (756): None	700 (93)	177	34.93	5.07	1	1	0.45
Yes	56 (7)	14	2.70	5.19	1.00 (0.34–2.97)	1.55 (0.50–4.81)	
4. Hookworm (747): None	705 (94)	185	35.18	5.26	1	1	0.05
Yes	42 (6)	4	1.99	2.01	0.37 (0.14–0.98)	0.33 (0.11–1.02)	
5.*M. perstans* (864): None	839 (97)	206	41.87	4.92	1	1	0.28
Yes	25 (3)	9	1.14	7.86	1.54 (0.66–3.59)	1.60 (0.68–3.77)	
Child's atopic status (assessed at 3 yr)§							
Skin prick responses (569)							
Negative	464 (82)	67	22.71	2.95	1	–	<0.0001
Positive	105 (18)	49	5.08	9.65	3.26 (1.84–5.80)	–	
Dermatophagoides-specific IgE (540)							
Non-atopic	478 (89)	93	23.34	3.98	1	–	0.21
Atopic	62 (11)	20	3.02	6.62	1.66 (0.75–3.69)	–	
Cockroach-specific IgE (540)							
Non-atopic	448 (83)	91	21.90	4.15	1	–	0.67
Atopic	92 (17)	22	4.46	4.94	1.18 (0.55–2.55)	–	

Pyrs, person years; CI, confidence interval; HR, hazard ratio; aHR, adjusted hazard ratio.

* For categorical variables, this is the p-value for the test of trend.

† Adjusted for maternal education, area of residence and household socio-economic status; childhood illnesses were additionally adjusted for the total number of clinic visits a child made during the study period. Breastfeeding was not adjusted for as 98% of the children in this study were breastfed.

‡ Children with data on worm infections at all five annual visits were included for this analysis.

§ Three-year-old children seen between November 2007 and March 2009 were selected for skin prick tests (4 allergens) and allergen-specific IgE testing.

No of missing variables:

¶ = 430,

∥ = 2.

Three-year-old children with a positive skin prick test (to any of the four allergens) had a higher incidence of eczema. Positive allergen-specific IgE responses to Dermatophagoides mix, and German cockroach allergens showed no overall association with eczema.

#### Effect modification of maternal hookworm on risk factors associated with eczema

To understand why the association between atopy and allergy is weak in developing countries, we examined the role of worms as potential effect modifiers. We investigated whether hookworm, the most prevalent maternal worm, modified the effects of atopy and other risk factors positively associated with eczema in this analysis (Table3). We found a strong positive association between maternal history of eczema and childhood eczema among children whose mothers had no hookworm infection during pregnancy, but not among children whose mothers were infected with hookworm (interaction p-value 0.05), despite similarities in the prevalence of history of eczema among mothers with or without hookworm. Similar trends towards effect modification by hookworm were observed for associations between eczema and maternal albendazole treatment during pregnancy and between eczema and skin prick test responses in the children, but these were not statistically significant. Female children had a higher eczema incidence if their mothers had no hookworm, but not if they had hookworm (interaction p-value 0.04). Dermatophagoides mix-specific IgE showed a strong positive association with eczema among the children whose mothers had no hookworm during pregnancy, but not among children whose mothers had hookworm (interaction p-value 0.03). Cockroach-specific IgE had a weak positive association with eczema among children whose mothers had no hookworm, but a weak inverse association among children whose mothers had hookworm (interaction p-value 0.01). Less prevalent maternal worm infections showed a similar trend to that of hookworm but power to detect effect modification was low (results not shown).

**Table 3:** The interaction of maternal hookworm infection during pregnancy with the other risk factors for eczema in the first five years of life

Maternal hookworm status	Number (%) with given risk factor	No. of eczema events	Person years at risk (×100)	Rate (per 100 pyrs)	Crude HR (95% CI)	aHR* (95% CI)	p-value	Test for interaction p-value
Maternal history of eczema (1766)								
None	No 955 (96)	240	44.33	5.41	1	1	0.008	0.05
	Yes 35 (4)	24	1.60	14.96	2.78 (1.29–6.00)	2.87 (1.31–6.27)		
Yes	No 751 (97)	131	34.42	3.81	1	1	0.60	
	Yes 21 (3)	3	0.95	3.17	0.83 (0.28–2.42)	0.73 (0.23–2.30)		
Maternal albendazole during pregnancy (2345)								
None	Placebo 643(49)	103	26.65	3.86	1	–	0.006	0.15
	Albendazole 668 (51)	195	27.68	7.04	1.82 (1.19–2.79)	–		
Yes	Placebo 528 (52)	72	21.41	3.36	1	–	0.56	
	Albendazole 497 (48)	79	20.37	3.88	1.15 (0.72–1.83)	–		
Child's gender (2334)								
None	Male 695 (53)	113	28.46	3.97	1	–	0.004	0.04
	Female 614 (47)	185	25.87	7.15	1.82 (1.22–2.73)	–		
Yes	Male 506 (49)	77	20.94	3.68	1	–	0.87	
	Female 519 (51)	74	20.84	3.55	0.96 (0.60–1.53)	–		
Child's skin prick test positive responses (569)†								
None	Negative 264 (79)	28	12.93	2.16	1	–	<0.0001	0.18
	Positive 70 (21)	35	3.41	10.27	4.74 (2.30–9.73)	–		
Yes	Negative 199 (85)	37	9.73	3.80	1	–	0.08	
	Positive 35 (15)	14	1.67	8.37	2.20 (0.92–5.26)	–		
Child's dermatophagoides mix-specific IgE responses (539)†								
None	Non-atopic 276 (87)	45	13.50	3.33	1	–	0.03	0.03
	Atopic 41 (13)	18	1.98	9.07	2.72 (1.11–6.63)	–		
Yes	Non-atopic 201 (91)	46	9.79	4.70	1	–	0.22	
	Atopic 21 (9)	2	1.04	1.93	0.41 (0.10–1.69)	–		
Child's German cockroach-specific IgE responses (539)†								
None	Non-atopic 258 (81)	43	12.64	3.40	1	–	0.10	0.01
	Atopic 59 (19)	20	2.84	7.04	2.06 (0.87–4.85)	–		
Yes	Non-atopic 189 (85)	46	9.21	4.99	1	–	0.06	
	Atopic 33 (15)	2	1.62	1.24	0.25 (0.06–1.04)	–		

Pyrs, person years; CI, confidence interval; HR, hazard ratio; aHR, adjusted hazard ratio.

* Adjusted for maternal education, area of residence and household socio-economic status.

† Three-year-old children seen between November 2007 and March 2009 were selected for r skin prick tests (4 allergens) and allergen-specific IgE testing.

#### Effect of trial anthelminthic drugs on the association between worms and eczema

We found a strong inverse association between maternal hookworm and eczema if the mother received albendazole [aHR (95% CI) 0.57 (0.37–0.90)] that was not present if the mother received albendazole placebo [0.99 (0.62–1.60)], although this interaction was not significant (p-value 0.13). The association between childhood hookworm (and*T. trichiura*) and eczema was not influenced by the three-monthly albendazole/placebo that the children were randomized to receive.

## Discussion

This is the first study to show that maternal hookworm infection is inversely associated with eczema incidence and modifies effects of known risk factors for childhood eczema such as mother's history of eczema, child's gender and atopic status.

We also found that early childhood hookworm and*T. trichiura*, although of low prevalence, were associated with a reduced incidence of eczema. It is important to note that most eczema episodes occurred in infancy, before most children acquired any worm infections, suggesting that the observed inverse association is not causal but may be due to other environmental exposures or to a genetic predisposition – such that children predisposed to eczema might be less susceptible to worm infections.

The study had a number of limitations. First, worm infections were determined by the Kato Katz method using a single stool sample, which is relatively insensitive (23). Secondly, confounding is an inherent flaw of cohort studies, and it is possible that there are unmeasured confounders. Thirdly, clinicians were not blinded to the worm status of the participants, which may have led to observer bias. However, this is unlikely to have affected the antibody results as immunology technicians were unaware of the worm status of the participants. Lastly, we tested only a sample of children for atopy. This reduced power of the study, particularly for interaction analyses.

Our findings are generalisable to other communities in developing countries with high prevalence but low intensity of hookworm infections.

Other studies have investigated possible modifying effects of worms on the association between atopy and allergy and obtained results similar to ours. A case–control study in Ecuador found that the association between atopy and wheeze was stronger among uninfected children compared with those with geohelminths (24). A study among Cuban children found a strong positive association between atopy and physician-diagnosed asthma among children without Toxocara antibodies, but a weak association among those with Toxocara antibodies (25). This evidence suggests that worm infections may, at least in part, explain why atopy is weakly associated with clinical allergy in developing countries, in contrast to the strong associations reported in developed countries.

The finding of an inverse association between malaria and eczema was unexpected but was consistent for asymptomatic malaria in pregnancy and for clinical malaria illnesses in early childhood. Malaria is a potent immune modulator which can influence the response to unrelated antigens (26).

The observation that children from rural areas were at reduced risk of eczema compared with their counterparts from urban areas has also been observed in other developing countries (27,28). This difference remained even after adjusting for worms and potential confounders. What is intriguing is that these urban/rural areas are located within the same district, a relatively small geographical area. Perhaps, multiple infections, or exposure to farm animals or other aspects of rural life, whose effect was not measured in this analysis, might explain these urban/rural differences.

This study has important implications for future research. First, there is urgent need to understand the underlying immune mechanisms by which hookworm could modify the effects of known risk factors for eczema. Such studies may take the form of clinical trials of worms for therapy or worm products. Indeed, early phase clinical trials, using iatrogenic hookworm, have been initiated in the United Kingdom (29,30). With careful attention to design, studies in developing countries can be useful as the effects of interventions for worm control on allergy are explored. Secondly, pregnancy and early childhood provide a window of opportunity during which interventions for the primary prevention of allergy could be targeted.

## Conclusion

We have demonstrated that prenatal hookworm exposure modifies the effects of known risk factors for childhood eczema and that early childhood worms are associated with reduced eczema incidence. More research is needed to understand possible underlying immune mechanisms and to explore the therapeutic potential of worms or their products, particularly during pregnancy or early childhood, in the primary prevention of allergy.
